# Association of glycogen synthase kinase-3β with cognitive impairment in type 2 diabetes patients: a six-year follow-up study

**DOI:** 10.3389/fendo.2024.1386773

**Published:** 2024-04-10

**Authors:** Wei Wei, Pan Xu, Li Li, Hong Mao, Na Li, Xiao-qing Wang, Li Wang, Zhi-peng Xu, Shi Zhao

**Affiliations:** ^1^ Department of Endocrinology, The Central Hospital of Wuhan, Tongji Medical College, Huazhong University of Science and Technology, Wuhan, China; ^2^ Department of Neurology, The Central Hospital of Wuhan, Tongji Medical College, Huazhong University of Science and Technology, Wuhan, China; ^3^ College of Medicine and Health Science, Wuhan Polytechnic University, Wuhan, China; ^4^ Department of Nursing, The Central Hospital of Wuhan, Tongji Medical College, Huazhong University of Science and Technology, Wuhan, China; ^5^ Ministry of Education Key Laboratory for Neurological Disorders, Hubei Key Laboratory for Neurological Disorders, Department of Pathophysiology, School of Basic Medicine, Tongji Medical College, Huazhong University of Science and Technology, Wuhan, China

**Keywords:** type 2 diabetes mellitus, mild cognitive impairment, glycogen synthase kinase-3β, ApoE gene, Alzheimer’s disease

## Abstract

**Background:**

Our previous multicenter case-control study showed that aging, up-regulation of platelet glycogen synthase kinase-3β (GSK-3β), impaired olfactory function, and ApoE ϵ4 genotype were associated with cognitive decline in type 2 diabetes mellitus (T2DM) patients. However, the causal relationship between these biomarkers and the development of cognitive decline in T2DM patients remains unclear.

**Methods:**

To further investigate this potential relationship, we designed a 6-year follow-up study in 273 T2DM patients with normal cognitive in our previous study. Baseline characteristics of the study population were compared between T2DM patients with and without incident mild cognitive impairment (MCI). We utilized Cox proportional hazard regression models to assess the risk of cognitive impairment associated with various baseline biomarkers. Receiver operating characteristic curves (ROC) were performed to evaluate the diagnostic accuracy of these biomarkers in predicting cognitive impairment.

**Results:**

During a median follow-up time of 6 years (with a range of 4 to 9 years), 40 patients (16.13%) with T2DM developed MCI. Participants who developed incident MCI were more likely to be older, have a lower education level, have more diabetic complications, a higher percentage of ApoE ϵ4 allele and a higher level of platelet GSK-3β activity (rGSK-3β) at baseline (*P<0.05*). In the longitudinal follow-up, individuals with higher levels of rGSK-3β were more likely to develop incident MCI, with an adjusted hazard ratio (HR) of 1.60 (95% confidence interval [CI] 1.05, 2.46), even after controlling for potential confounders. The AUC of the combination of age, rGSK-3β and ApoEϵ4 allele predicted for incident MCI was 0.71.

**Conclusion:**

Platelet GSK-3β activity could be a useful biomarker to predict cognitive decline, suggesting the feasibility of identifying vulnerable population and implementing early prevention for dementia.

## 1 Introduction

Alzheimer’s disease (AD) is a progressive neurodegenerative disease characterized by gradual decline of cognitive functions, which affects memory, thinking, and behavior (1). It is one of the most common causes of dementia, affecting millions of people worldwide. Currently, the pathogenesis of AD is still not fully understood. The abnormal aggregation and deposition of Aβ protein in the brain is one of the main pathological features of AD, causing neuronal damage and death. The hyperphosphorylation of tau protein can also lead to the formation of neurofibrillary tangles, which is another important pathological feature of AD (2, 3). Over the past decade, China has experienced a remarkable increase in the occurrence of type-2 diabetes mellitus (T2DM), with an elevated prevalence of 11.2% among adults aged 18 and above. Furthermore, the elderly population aged 70 and above exhibits an even more concerning trend, with a prevalence rate of 28.8% (4). T2DM may contribute to the development of AD through multiple common mechanisms involving insulin resistance (5), inflammation, advanced glycation end-products (AGEs), vascular disease and lifestyle choices (6). The increase in the aging population in China has led to a rise in the prevalence of AD and T2DM. These diseases exert a profound impact on the well-being of the elderly population and present a significant and escalating financial and healthcare burden on the country, making their prevention and management a top priority.

Epidemiological studies have established T2DM as an independent risk factor for mild cognitive impairment (MCI), a condition that often serves as a precursor to AD (7). MCI is a condition in which individuals experience slight but noticeable declines in cognitive functions such as memory, attention, and executive functions. Although these declines are not severe enough to be diagnosed as dementia, they can significantly impact a person’s daily life and are often precursors to AD (8). Given the absence of effective drug treatments for MCI, preventative measures have become paramount in preserving cognitive function. Therefore, it is essential to identify individuals at increased risk of developing MCI among the growing population with T2DM to enable timely intervention and potentially delay the onset of AD.

In recent years, there has been a growing body of evidence that strongly suggests the potential role of glycogen synthase kinase-3β (GSK-3β) as a link between T2DM and MCI (9). GSK-3β is a crucial kinase that contributes to the generation of Aβ, tau hyperphosphorylation, and long-term synaptic inhibition, which are observed in both AD and T2DM (10). However, the exact association between MCI and T2DM remains unclear. And the preliminary findings are based on limited data obtained from cross-sectional studies with heterogeneous study populations and measurements of cognitive function.

Our previous study has found that aging, increased activity of GSK-3β, the presence of ApoE ϵ4 genotype, and olfactory dysfunction are associated with cognitive decline in T2DM patients (11). To prospectively confirm this relationship, we designed a 6-year follow-up study involving 273 T2DM patients without MCI in our previous study. To the best of our knowledge, this is the first longitudinal study to investigate the association between peripheral biomarkers and early mild cognitive decline in a T2DM cohort.

## 2 Materials and methods

### 2.1 Study design and participants

As one of five medical centers, we conducted a follow-up study in the cohort population from our previous multi-centre, retrospective, nested case-control study aimed at evaluating peripheral biomarkers for diagnosing MCI in T2DM.

Briefly, between January 2012 and May 2015, 341 patients with T2DM from Wuhan Central Hospital were included in the study. At the beginning of the study, each participant underwent a comprehensive evaluation, including neuropsychological evaluation, assessment of olfactory function, ApoE genotyping and measurement of platelet GSK-3β activity. The details of this evaluation have been described in previous reports (11). To determine the activity levels of GSK-3β, the total GSK-3β (tGSK-3β) and serine-9 phosphorylated GSK-3β (pS9GSK-3β, the inactive form of the kinase) were measured in platelets, using an enzyme activity assay kit. The ratio of tGSK-3β to pS9GSK-3β (rGSK-3β) was used as a surrogate marker of platelet GSK-3β activity.

At baseline and follow-up interview, demographic data including age, sex, education, cigarette smoking, and habitual alcohol consumption were collected. The level of education was determined by the maximum years of formal education and categorized as≤ 6 years (primary school), 7-9 (middle school) and ≥10 years (high school or college). Body mass index (BMI) is calculated by dividing weight (kg) by the square of height (m). The medical history of diabetic complications, diabetic treatment, hypertension, hyperlipidemia, and cardiovascular disease were collected by self-report from participants or reviewed through the hospital information system.

**Figure 1:**
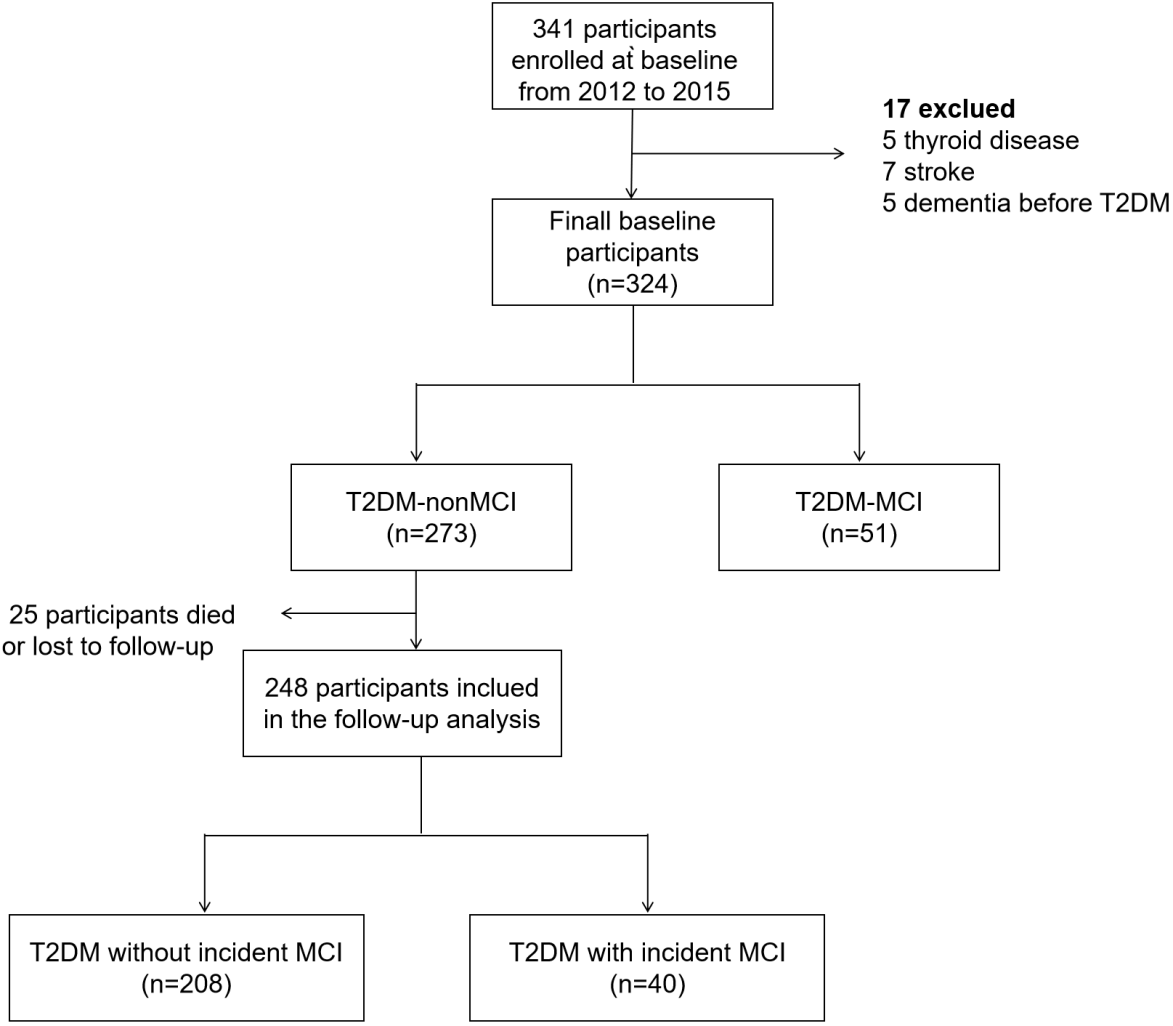
Study flowchart.

The participants diagnosed with MCI at baseline enrollment were excluded from further assessment. Therefore, a total of 273 T2DM patients without MCI at baseline were invited to participate in the second round of neuropsychological evaluation from December 2021 to August 2022. During the follow-up period, 27 participants either passed away or withdrew from the study, leaving a subsample of 246 patients with T2DM for final analysis (**Figure 1**). As required by the Declaration of Helsinki, all participants provided written informed consent prior to the second round of neuropsychological assessment. The second phase of the follow-up study received ethical approval from the Ethics Committee of Wuhan Central Hospital.

### 2.2 Diagnosis of incident MCI

The neuropsychological assessment mainly consisted of the translated version of Minimum Mental State Examination (MMSE) and Clinical Dementia Rating (CDR). These assessments were conducted by two examiners with neurological training and experience in neurophysiologic techniques.

The diagnosis of MCI was based on Petersen’s MCI criteria (12): 1.difficulties in subjective memory and cognitive; 2. objective cognitive impairment with the MMSE score below 27 or education-adjusted mean values; 3. CDR score≥0.5.

Incident MCI was determined in subjects who developed MCI before the second round of neuropsychological evaluation. Specifically, we divided the outcome at follow-up into 2 categories: remaining with normal cognition (T2DM-NC) and progressing to MCI (T2DM-CI).

### 2.3 Statistical analysis

Data analysis was conducted using IBM’s SPSS Statistics 26.0 software. For continuous variables with a normal distribution, mean ± standard deviation (SD) was used to present the data. For skewed variables, the median (inter-quartile range) was reported. Categorical variables were presented as frequency (%). To compare categorical and continuous variables between case and control groups, Chi square and t-test tests were used, respectively. All P values were statistically significant if P< 0.05.

Baseline characteristics of the study population were compared between T2DM patients who developed MCI and those who did not, using statistical methods such as covariance analysis, chi-squared tests, and two-sample t-tests. Spearman’s correlation analysis was utilized to assess relationships between rGSK-3β and continuous covariates. To determine the incidence rate of MCI during the follow-up period, the number of new cases of MCI observed was divided by 1000 person-years. The person-years were calculated as the time from enrollment to either the end of follow-up or the development of MCI, whichever occurred first. The Kaplan-Meier curve with log-rank test was utilized to visualize and statistically analyze the incidence of MCI in individuals with and without the ApoEϵ4 allele.

The cox proportional hazard regression models were used to assess the risk of cognitive impairment during follow-up associated with 1-value increment of baseline rGSK-3β, using hazard ratios (HRs) along with 95% confidence interval (95%CI). Model 1 was a crude model. Model 2 adjusted for potential confounders, including age, sex, BMI, smoking, habitual alcohol consumption, education level, diabetic therapy, duration of diabetes, diabetic complications, cardiovascular disease, hypertension, hyperlipidemia, glycosylated hemoglobin A1c (HbA1c) and fasting plasma glucose (FPG), and Model 3 further adjusted for ApoE genotyping and the olfactory score.

Binary logistic regression models were constructed to test whether the biomarkers in our previous study were associated with the diagnosis of incident MCI during follow-up. The odds ratio (OR) and 95% CI were calculated for incident MCI of these biomarkers. The optimal cut-off value for each biomarker, providing the best combination of sensitivity and specificity, was determined. The area under curve (AUC) and diagnostic accuracy were also calculated. Receiver operating characteristic (ROC) curves were plotted to evaluate the diagnostic accuracy of rGSK-3β and the combined biomarkers.

## 3 Results

### 3.1 Demographics and clinical characteristics

Between 2012 and 2015, we conducted a screening of 341 participants, of whom 324 met the inclusion criteria. The descriptive statistics based on the baseline neuropsychological evaluation are provided in **Supplementary Table 1**. In consist with our previous study, individuals in T2DM-MCI group exhibited older age, lower MMSE score, lower education level, more diabetic complications, worse olfactory function, and higher fasting blood glucose (FPG) levels compared to the T2DM-nMCI group (*P<0.05*). Additionally, the proportion of ApoEϵ4 allele was higher in the T2DM-MCI group than in the T2DM-nMCI group (*P<0.05*).

Participants diagnosed with MCI at baseline were excluded from further evaluation. Among 273 patients in T2DM -MCI group, 27 participants either died or were lost to follow-up. A comparison of baseline characteristics revealed a modest difference between participants who attended the follow-up study (attenders) and those who did not (non-attenders) (**Supplementary Table 2**).

### 3.2 Associations between baseline rGSK-3β and cognitive impairment

**Figure 2:**
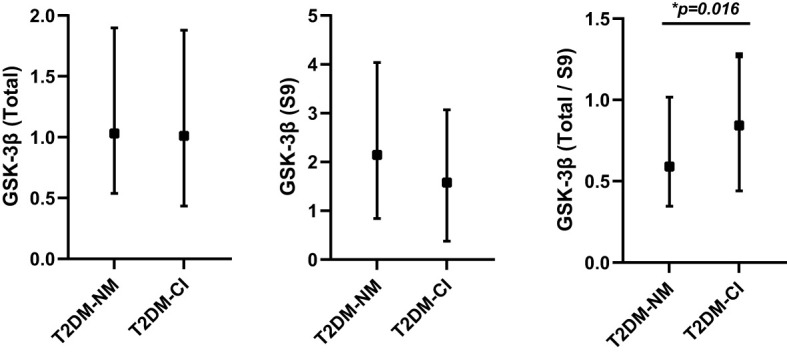
The differences of total GSK-3β, pS9 GSK-3β and rGSK-3β between T2DM patients with incident MCI (T2DM-CI; n=40) and without incident MCI (T2DM-NM; n=208). *P<0.05, T2DM-NM group versus the T2DM-CI group. GSK-3β, glycogen synthase kinase-3β; pS9GSK-3β, serine-9 phosphorylated GSK-3β; rGSK-3β, total GSK-3β/serine-9 phosphorylated GSK-3β.

Over a median follow-up time of 6 years (ranging from 4 to 9 years), 40 (16.13%) of 248 patients with T2DM developed MCI. The overall incidence of MCI during the follow-up period was 24.7 per 1000 patient-years. **Table 1** represented the baseline characteristics of the study population between incident MCI (T2DM-CI) and cognitively normal (T2DM-NC). Participants with incident MCI at baseline were found to be older, less educated, had more diabetic complications, a higher percentage of ApoEϵ4 allele and higher levels of rGSK-3β (all *P <0.05*, **Figure 2**).

**Table 1:** Baseline characteristics between T2DM patients with and without incident MCI.

	T2DM-NM	T2DM-CI	
	(n=208)	(n=40)	P value
MMSE at follow-up	28.34 ± 0.94	24.48 ± 2.00	***<0.001*** **
MMSE at baseline	28.86 ± 1.04	28.35 ± 1.03	***0.005*** **
Years of follow-up (years)	6.52 ± 0.93	6.55 ± 0.88	0.859
Age (years)	62.34 ± 6.86	67.03 ± 8.82	***0.003*** **
Male (%)	85 (40.87%)	21 (52.50%)	0.222
BMI (kg/m²)	24.17 ± 2.81	25.06 ± 3.66	0.154
Cigarette smoking (%)	33 (15.87%)	6 (15.00%)	1.000
Habitual alcohol drinking (%)	17 (8.17%)	3 (7.50%)	1.000
**Education**			***0.044*** *
≤ 6 years (Primary school)	27 (12.98%)	11 (27.50%)	
7-9 (Middle school)	142 (68.27%)	25 (62.50%)	
≥ 10 years (High school or college)	39 (18.75%)	4 (10.00%)	
Oral medication only (%)	143 (68.75%)	25 (62.50%)	0.463
Insulin (%)	82 (39.42%)	18 (45.00%)	0.598
Duration of diabetes (years)	7.69 ± 5.71	8.76 ± 7.18	0.299
Diabetic complications (%)	81 (38.94%)	23 (57.50%)	***0.036*** *
Diabetic Retinopathy (%)	44 (21.15%)	13 (32.50%)	0.150
Diabetic Nephropathy (%)	16 (7.69%)	9 (22.50%)	***0.009*** **
Diabetic Peripheral Neuropathy (%)	38 (18.27%)	6 (15.00%)	0.821
Cardiovascular disease (%)	23 (11.06%)	5 (12.50%)	0.786
Hypertension (%)	105 (50.48%)	24 (60.00%)	0.303
Hyperlipidemia (%)	46 (22.12%)	8 (20.00%)	0.838
HbA1c (%)	7.77 ± 1.74	7.51 ± 1.30	0.367
FPG (mmol/L)	8.28 ± 3.01	8.24 ± 2.80	0.927
Olfactory	6.94 ± 1.72	6.90 ± 1.83	0.894
ApoE ϵ2	47 (22.60%)	9 (22.50%)	1.000
ApoE ϵ3	194 (93.27%)	36 (90.00%)	0.504
ApoE ϵ4	21 (10.10%)	11 (27.50%)	***0.008*** **
tGSK-3β	1.03 (0.54 - 1.90)	1.07 (0.44 - 2.47)	0.286
pS9GSK-3β	2.14 (0.84 - 4.04)	1.58 (0.40 - 3.18)	0.194
rGSK-3β	0.59 (0.35 - 1.02)	0.84 (0.44- 1.27)	***0.016*** *

*, p value<0.05; **,p value<0.01.

T2DM, type-2 diabetes mellitus; MCI, mild cognitive impairment; T2DM-NM,T2DM patients remaining with normal cognition; T2DM-CI, T2DM patients progressing to MCI; MMSE, Minimum Mental State Examination; BMI, body mass index; FPG, fasting plasma glucose; HbA1c, glycosylated hemoglobin A1c; ApoE, apolipoprotein E; GSK-3β, glycogen synthase kinase-3β; tGSK-3β, total GSK-3β; pS9GSK-3β, serine-9 phosphorylated GSK-3β; rGSK-3β, total GSK-3β/Ser9 GSK-3β.Bold values indicate statistical significance.

**Figure 3:**
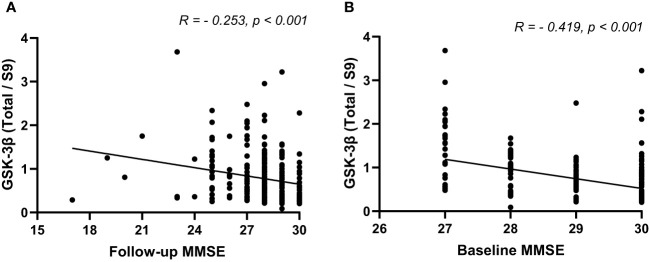
The Spearman’s correlation analysis between rGSK-3β and baseline MMSE **(A)** and follow-up MMSE **(B)**. MMSE, Minimum Mental State Examination; rGSK-3β, total GSK-3β/serine-9 phosphorylated GSK-3β.

**Figure 4:**
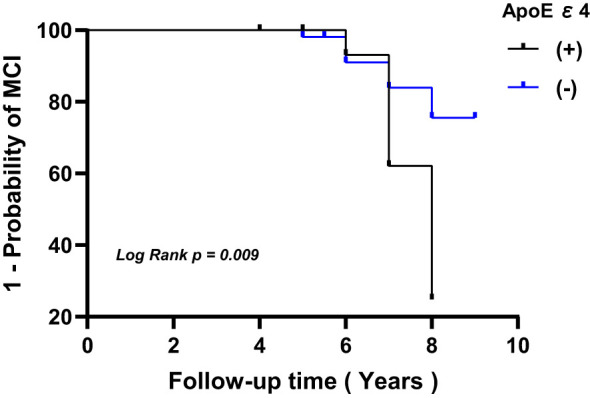
Kaplan-Meier survival curves estimating for the development of MCI based on the presence of ApoEϵ4 allele. MCI, mild cognitive impairment; ApoE, apolipoprotein E.

In patients with T2DM, the levels of rGSK-3β were inversely related to MMSE scores both at the initial evaluation and at follow-up (both P<0.001, **Figure 3**). **Figure 4** depicts Kaplan-Meier survival curves estimating for the development of MCI based on the presence of ApoEϵ4 allele. T2DM patients without ApoEϵ4 allele had a lower survival rate without MCI (P <0.05 by log-rank statistics).

As shown in **Table 2**, participants with higher levels of rGSK-3β at baseline had a significantly higher risk of developing MCI. For each 1-unit increase in rGSK-3β, the HRs for incident MCI were 1.60 (95% CI 1.05, 2.46). This association was further strengthened by adjustments for sociodemographic variables, ApoE4 genotypes and olfactory scores.

**Table 2:** Longitudinal association of baseline rGSK-3β with incident MCI among individuals with T2DM and normal cognition at baseline.

Variable	B	*S.E.*	Wald	*Sig.*	Exp(B)	95% CI	
						Lower	Upper
Modle 1							
rGSK-3β	0.472	0.217	4.710	0.030	1.603	1.047	2.455
Modle 2							
Age	0.060	0.018	10.613	0.001	1.062	1.023	1.101
rGSK-3β	0.416	0.200	4.332	0.037	1.516	1.025	2.243
Modle 3							
Age	0.089	0.019	21.48	0.000	1.093	1.052	1.134
rGSK-3β	0.651	0.258	6.372	0.012	1.917	1.157	3.176

Model 1 was a crude model.

Model 2 was adjusted for potential confounding factors, including age, sex, body mass index (BMI), smoking, habitual alcohol consumption, education level, diabetes therapy, duration of diabetes, diabetic complications, cardiovascular disease, hypertension, hyperlipidemia, glycosylated hemoglobin A1c (HbA1c) and fasting plasma glucose (FPG).

Model 3 was adjusted for the variables in model 2 plus ApoE genotyping and the olfactory score.

GSK-3β, glycogen synthase kinase-3β;rGSK-3β, total GSK-3β/serine-9 phosphorylated GSK-3β.

### 3.3 Diagnostic efficacy of single biomarker and the combined biomarkers in predicting MCI in T2DM patients

We have previously observed that cognitive decline in patients with T2DM is associated with advanced age, impaired olfactory function, increased platelet GSK-3β activity, and ApoE4 genotype. To investigate whether these biomarkers can predict the development of MCI during follow-up, we conducted binary logistic regression analysis.

Our results indicated that age (OR 1.09, 95% CI 1.04, 1.14), ApoE4 genotype (OR 2.68, 95% CI 1.11, 6.47), platelet GSK-3β activity (OR 1.87, 95% CI 1.05, 3.34) are independently associated with cognitive decline at follow-up. However, no significant association was observed for olfactory score. Therefore, we focused our subsequent analysis on these three factors and their combinations.

**Figure 5:**
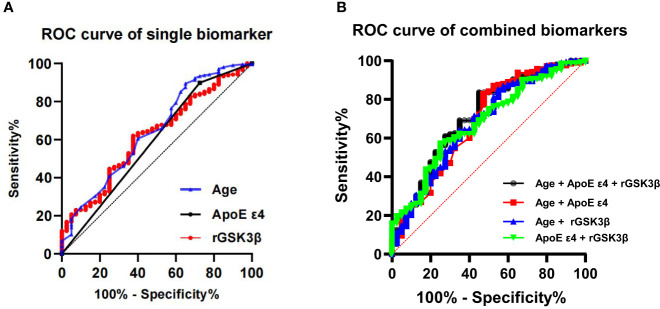
ROC show diagnostic efficacy of the single **(A)** and combined biomarkers **(B)** in predicting incident MCI in T2DM patients. ROC, receiver operating curves; ApoE, apolipoprotein E; GSK-3β, glycogen synthase kinase-3β; rGSK-3β, total GSK-3β/serine-9 phosphorylated GSK-3β.

We applied ROC models to calculate the AUC, accuracy, specificity and sensitivity of single biomarker of age, ApoE4 genotype and rGSK-3β for diagnosing incident MCI in T2DM patients (**Figure 5**) Among the three biomarkers, age exhibited a maximum AUC of 64% and an accuracy of 80.6% (**Table 3**). The ApoE4 genotype had a maximum AUC of 59% and an accuracy of 79.8%, while platelet GSK-3β activity showed a maximum AUC of 62% and an accuracy of 62.1%. By combining age, rGSK-3β and ApoEϵ4, we created a ROC curve with a maximum AUC of 71% and an accuracy of 79% (**Figure 5**; **Table 3**). When only age and ApoEϵ4 were considered, the AUC was 68% and the accuracy was 77.8%. When age and rGSK-3βwere combined, the maximum AUC was 68% and the accuracy was 78.6%. Combining ApoEϵ4 and rGSK-3βresulted in a maximum AUC of 68% and an accuracy of 60.1%.

**Table 3:** The diagnostic efficacy of Single Biomarker and the Combined Biomarkers in predicting MCI in T2DM patients.

Variables	Cutoff	Specificity	Sensitivity	AUC (95% CI)	Accuracy
Age	70.5	0.894	0.350	0.64 (0.55, 0.74)	0.806
ApoE ϵ4	–	0.899	0.275	0.59 (0.48, 0.69)	0.798
rGSK3β	0.79	0.620	0.625	0.62 (0.53, 0.71)	0.621
Age + ApoE ϵ4 + rGSK3β	–	0.837	0.550	**0.71 (0.62, 0.83)**	0.790
Age + ApoE ϵ4	–	0.827	0.525	0.68 (0.59, 0.78)	0.778
Age + rGSK3β	–	0.851	0.450	0.68 (0.59, 0.78)	0.786
ApoE ϵ4 + rGSK3β	–	0.572	0.750	0.68 (0.59, 0.77)	0.601

ROC, receiver operating characteristics; AUC, the area under the curve; CI, confidence interval;

GSK-3β, glycogen synthase kinase-3β; rGSK-3β, total GSK-3β/serine-9 phosphorylated GSK-3β.

## 4 Discussion

Alzheimer’s Disease (AD) and Type 2 Diabetes Mellitus (T2DM) are two widespread chronic diseases that pose significant health challenges to individuals and healthcare systems (13), particularly as the global population continues to age. There is a growing body of evidence suggesting that T2DM is associated with an increased risk of developing AD (7). Mild Cognitive Impairment (MCI) is often a precursor to AD. Early diagnosis and intervention are critical for improving outcomes and quality of life for individuals with MCI and reducing the risk of progression to AD. Currently, reliable biomarkers for early detection of cognitive impairment are lacking. Therefore, searching for a dependable set of biomarkers that can identify patients at high risk of cognitive decline is crucial to enabling early intervention and preventing the severe decline of cognitive function.

In our previous multicenter case-control study, we observed a strong correlation between platelet GSK-3β activation and T2DM patients with MCI. In continuation of our previous research, we have discovered that T2DM patients with normal cognition at baseline with higher levels of platelet GSK-3β activation were more likely to develop incident MCI during follow-up. This increased risk remained statistically significant even after adjusting for potential confounding factors, such as ApoE ϵ4 allele and impaired olfactory function.

GSK-3β is a conserved serine/threonine kinase that regulates multiple signaling pathways, there by influencing many critical cellular processes (14). Its activity can be increased by phosphorylation at Tyr 216 site and decreased by phosphorylation at Ser 9 site (15). GSK-3β is an essential kinase in the insulin pathway that serves as a vital link between the pathologies of T2DM and dementia (16). Studies have shown that the dysregulation of GSK-3β is associated with the development of insulin deficiency and insulin resistance (16, 17), as well as abnormal tau phosphorylation leading to the toxicity of neurofibrillary tangles (NFT) (10). Early alterations of tau protein may result in significant cognitive abnormalities (18). Additionally, GSK-3β has been implicated in the pathogenesis of several neurodegenerative diseases, including Alzheimer’s disease, Parkinson’s disease, and mood disorders. GSK-3β activity could be a valuable biomarker for diagnosing and potentially monitoring the progression of these neurological conditions (19).

Clinical studies have also revealed a strong correlation between excessive activity of GSK-3β and cognitive impairment (9, 20). Despite the considerable number of studies have investigated the correlation between GSK-3β and cognitive function, the findings have been inconclusive. Most of these studies were conducted utilizing a cross-sectional design, which precludes the establishment of a causal relationship between GSK-3β and MCI. In the current study, we provided a perspective view on changes in cognition by using the same cohort population with longitudinal data.

As GSK-3β has extensive substrates and biological functions, targeting GSK-3β may cause significant adverse effects. However, a simple and well-repeated protocol for measuring GSK-3β activation could serve as a convenient and cost-effective tool for early detection of MCI in patients with T2DM. Due to the current unavailability of brain or cerebrospinal fluid samples from these T2DM patients, it is not feasible to ascertain whether the activation of platelet GSK-3β reflects GSK-3β activation in central nervous system, nor can we provide direct evidence revealing the possible biological relationship between platelet GSK-3β and cognitive impairment. Platelets share homeostatic features with neurons, making them an attractive model for studying metabolic abnormalities in AD (21, 22). Further research is needed to establish the relationship between platelet GSK-3β activation and brain GSK-3β activation, as well as its biological relations with cognitive impairment. The necessity to verify the accuracy, sensitivity, and specificity of platelet GSK-3β activation as a predictive biomarker for cognitive impairment, as well as its potential application in a broader range of diseases, needs to be verifying in more large-scale, longitudinal studies.

Furthermore, aging is recognized as a critical factor in the development of AD. Our previous research has demonstrated that aging, ApoE ϵ4 allele and decline in olfactory function may serve as indicators of cognitive impairment in T2DM patients. Further analysis showed that the presence of the ApoEϵ4 allele in T2DM patients may interact with increased GSK-3β activity to accelerate cognitive decline (23). However, our current study has revealed that ApoE ϵ4 allele and olfactory decline did not predict cognitive decline, indicating their limited clinical utility. Therefore, it is imperative for future studies to investigate the impact of ApoE ϵ4 genotype and olfactory dysfunction on the onset of cognitive impairment in a larger population with a longer follow-up period.

We also investigated the diagnostic accuracy of age, ApoE ϵ4 genotype and rGSK-3β. Age exhibited the highest AUC and accuracy among these three biomarkers. When these biomarkers were combined, the diagnostic accuracy improved to 0.71. Therefore, a well-established model integrating aging, ApoE ϵ4 genotype and elevated platelet GSK-3β activities has great potential to predict the development of cognitive impairment in patients with T2DM.

Diabetes complications and MCI are closely linked, with diabetes being a significant risk factor for the development of cognitive decline. In this study, we found patients with more diabetic complication, particularly diabetic nephropathy, will more likely to develop incident MCI during follow-up. Both diabetic nephropathy and cognitive impairment share common risk factors such as poor glycemic control, hypertension, lymphatic dysfunction and dyslipidemia (24, 25). Therefore, the results of this study implied the importance of strengthening the management of diabetic complications to prevent or delay the onset of cognitive impairment in clinical practice.

There are several limitations in our study. Firstly, the sample size was moderate, and the follow-up study was limited to our hospital. Secondly, the participants in our study were relatively young compared to other studies, which may result in observing less cognitive impairment. Some participants may develop MCI with longer follow-up. Thirdly, the diagnosis of MCI using MMSE scores may be subject to the subjective judgment of doctors, and may also be influenced by the education level and socioeconomic status of participants. However, in this follow-up study, we used education-adjusted MMSE scores to diagnose MCI.

In summary, we provided some evidence that platelet GSK-3β activity could be a useful biomarker to indicate T2DM patients who may develop MCI using the population from our previous cross-sectional study. It is applicable for early detection and timely management of MCI in a rapidly growing population of T2DM patients, potentially leading to a reduction in the prevalence of AD.

## Data availability statement

The original contributions presented in the study are included in the article/**Supplementary Material**. Further inquiries can be directed to the corresponding author.

## Ethics statement

The studies involving humans were approved by the Ethics Committee of Wuhan Central Hospital. The studies were conducted in accordance with the local legislation and institutional requirements. The participants provided their written informed consent to participate in this study. Written informed consent was obtained from the individual(s) for the publication of any potentially identifiable images or data included in this article.

## Author contributions

WW: Data curation, Methodology, Writing – original draft. PX: Data curation, Methodology, Writing – review & editing. LL: Data curation, Methodology, Writing – review & editing. HM: Data curation, Funding acquisition, Supervision, Writing – review & editing. NL: Writing – review & editing. X-QW: Data curation, Writing – review & editing. LW: Data curation, Writing – review & editing. Z-PX: Data curation, Methodology, Writing – review & editing. SZ: Conceptualization, Methodology, Project administration, Supervision, Writing – review & editing.

## Supplementary material

Supplementary Table 1 Baseline characteristics of T2DM patients with MCI and without MCI at baseline.

Supplementary Table 2 Baseline characteristics of T2DM patients attended and non-attended the follow-up study.

## References

[1] McKhann GM Knopman DS Chertkow H Hyman BT Jack CR Jr Kawas CH. The diagnosis of dementia due to Alzheimer's disease: recommendations from the National Institute on Aging-Alzheimer's Association workgroups on diagnostic guidelines for Alzheimer's disease. Alzheimers Dement. (2011) 7:263–9. doi: 10.1016/j.jalz.2011.03.005 PMC3312024 21514250

[2] Gasecka A Siwik D Gajewska M Jaguszewski MJ Mazurek T Filipiak KJ. Early biomarkers of neurodegenerative and neurovascular disorders in diabetes. J Clin Med. (2020) 9:2807. doi: 10.3390/jcm9092807 32872672 PMC7564566

[3] Thomas KR Bangen KJ Weigand AJ Edmonds EC Sundermann E Wong CG. Type 2 diabetes interacts with alzheimer disease risk factors to predict functional decline. Alzheimer Dis Assoc Disord. (2020) 34:10–7. doi: 10.1097/WAD.0000000000000332 PMC6952586 31305320

[4] Li Y Teng D Shi X Qin G Qin Y Quan H. Prevalence of diabetes recorded in mainland China using 2018 diagnostic criteria from the American Diabetes Association: national cross sectional study. BMJ. (2020) 369:m997. doi: 10.1136/bmj.m997 32345662 PMC7186854

[5] Arnold SE Arvanitakis Z Macauley-Rambach SL Koenig AM Wang HY Ahima RS. Brain insulin resistance in type 2 diabetes and Alzheimer disease: concepts and conundrums. Nat Rev Neurol. (2018) 14:168–81. doi: 10.1038/nrneurol.2017.185 PMC6098968 29377010

[6] Chen Y Yu Q Gong CX. Molecular connection between diabetes and dementia. Adv Exp Med Biol. (2019) 1128:103–31. doi: 10.1007/978-981-13-3540-2_6 31062327

[7] Xue M Xu W Ou YN Cao XP Tan MS Tan L. Diabetes mellitus and risks of cognitive impairment and dementia: A systematic review and meta-analysis of 144 prospective studies. Ageing Res Rev. (2019) 55:100944. doi: 10.1016/j.arr.2019.100944 31430566

[8] Biessels GJ Despa F. Cognitive decline and dementia in diabetes mellitus: mechanisms and clinical implications. Nat Rev Endocrinol. (2018) 14:591–604. doi: 10.1038/s41574-018-0048-7 30022099 PMC6397437

[9] Du B Lian Y Chen C Zhang H Bi Y Fan C. Strong association of serum GSK-3β/BDNF ratio with mild cognitive impairment in elderly type 2 diabetic patients. Curr Alzheimer Res. (2019) 16:1151–60. doi: 10.2174/1567205016666190827112546 31453785

[10] Kandimalla R Thirumala V Reddy PH. Is Alzheimer's disease a Type 3 Diabetes? A critical appraisal. Biochim Biophys Acta Mol Basis Dis. (2017) 1863:1078–89. doi: 10.1016/j.bbadis.2016.08.018 PMC5344773 27567931

[11] Xu ZP Yang SL Zhao S Zheng CH Li HH Wang JZ. Biomarkers for early diagnostic of mild cognitive impairment in type-2 diabetes patients: A multicentre, retrospective, nested case-control study. EBioMedicine. (2016) 6:105–13. doi: 10.1016/j.ebiom.2016.02.014 PMC4816853 27077117

[12] Petersen RC Smith GE Waring SC Ivnik RJ Tangalos EG Kokmen E. Mild cognitive impairment: clinical characterization and outcome. Arch Neurol. (1999) 56:303–8. doi: 10.1001/archneur.56.3.303 10190820

[13] Li K Wei S Liu Z Hu L Lin J Tan S. The prevalence of alzheimer's disease in China: A systematic review and meta-analysis. Iran J Public Health. (2018) 47:1615–26. PMC6294855 30581776

[14] Doble BW Woodgett JR. Role of glycogen synthase kinase-3 in cell fate and epithelial-mesenchymal transitions. Cells Tissues Organs. (2007) 185:73–84. doi: 10.1159/000101306 17587811

[15] Dajani R Fraser E Roe SM Young N Good V Dale TC. Crystal structure of glycogen synthase kinase 3 beta: structural basis for phosphate-primed substrate specificity and autoinhibition. Cell. (2001) 105:721–32. doi: 10.1016/s0092-8674(01)00374-9 11440715

[16] Zhang Y Huang NQ Yan F Jin H Zhou SY Shi JS. Diabetes mellitus and Alzheimer's disease: GSK-3β as a potential link. Behav Brain Res. (2018) 339:57–65. doi: 10.1016/j.bbr.2017.11.015 29158110

[17] Jayaraj RL Azimullah S Beiram R. Diabetes as a risk factor for Alzheimer's disease in the Middle East and its shared pathological mediators. Saudi J Biol Sci. (2020) 27:736–50. doi: 10.1016/j.sjbs.2019.12.028 PMC6997863 32210695

[18] Hochgräfe K Sydow A Mandelkow EM. Regulatable transgenic mouse models of Alzheimer disease: onset, reversibility and spreading of Tau pathology. FEBS J. (2013) 280:4371–81. doi: 10.1111/febs.12250 23517246

[19] Lai S Wang P Gong J Zhang S. New insights into the role of GSK-3β in the brain: from neurodegenerative disease to tumorigenesis. PeerJ. (2023) 11:e16635. doi: 10.7717/peerj.16635 38107562 PMC10722984

[20] Kumari S Singh A Singh AK Yadav Y Bajpai S Kumar P. Circulatory GSK-3β: blood-based biomarker and therapeutic target for alzheimer's disease. J Alzheimers Dis. (2022) 85:249–60. doi: 10.3233/JAD-215347 34776454

[21] Talib LL Joaquim HP Forlenza OV. Platelet biomarkers in Alzheimer's disease. World J Psychiatry. (2012) 2:95–101. doi: 10.5498/wjp.v2.i6.95 24175175 PMC3782189

[22] Forlenza OV Torres CA Talib LL de Paula VJ Joaquim HP Diniz BS. Increased platelet GSK3B activity in patients with mild cognitive impairment and Alzheimer's disease. J Psychiatr Res. (2011) 45:220–4. doi: 10.1016/j.jpsychires.2010.06.002 20576277

[23] Gao Y Yu H Liu Y Xu Z He B Liu H. GSK-3β activation mediates apolipoprotein E4-associated cognitive impairment in type 2 diabetes mellitus: A multicenter, cross-sectional study. J Diabetes. (2024) 16:e13470. doi: 10.1111/1753-0407.13470 37700547 PMC10809305

[24] Maimaitituerxun R Chen W Xiang J Xie Y Xiao F Wu XY. Predictive model for identifying mild cognitive impairment in patients with type 2 diabetes mellitus: A CHAID decision tree analysis. Brain Behav. (2024) 14:e3456. doi: 10.1002/brb3.3456 38450963 PMC10918605

[25] Drew DA Weiner DE Sarnak MJ. Cognitive impairment in CKD: pathophysiology, management, and prevention. Am J Kidney Dis. (2019) 74:782–90. doi: 10.1053/j.ajkd.2019.05.017 PMC7038648 31378643

