# Supplementary material for: Association of glycogen synthase kinase-3β with cognitive impairment in type 2 diabetes patients: a six-year follow-up study
Source: Front Endocrinol (Lausanne). 2024 Apr 10;15:1386773. doi: 10.3389/fendo.2024.1386773 (PMC11039938; doi:10.3389/fendo.2024.1386773)
Supplement: Supplementary Table 1 — Baseline characteristics of T2DM patients with MCI and without MCI at baseline. [file Table_1.docx]

**Supplementary Table 1. Baseline characteristics of T2DM patients with MCI and without MCI at baseline**

|  | **T2DM-nMCI** | **T2DM-MCI** |  |
| --- | --- | --- | --- |
| **Characteristics** | **(n=273)** | **(n=51)** | ***p* Value** |
| Age(years) | 63.48 ± 7.85 | 68.39 ± 8.45 | ***0.000*** |
| Male (%) | 117 (42.86%) | 21 (41.18%) | 0.878 |
| BMI (kg/m²) | 24.31 ± 2.94 | 25.40 ± 2.63 | 0.339 |
| Cigarette smoking (%) | 42 (15.38%) | 8 (15.69%) | 1.000 |
| Habitual alcohol drinking (%) | 23 (8.42%) | 3 (5.88%) | 0.779 |
| **Education** |  |  | ***0.044*** |
| ≤ 6 years (Primary school) | 43 (15.75%) | 11 (21.57%) |  |
| 7-9 (Middle school) | 183 (67.03%) | 38 (74.51%) |  |
| ≥ 10 years (High school or college) | 47 (17.22%) | 2 (3.92%) |  |
| Oral medication only (%) | 186 (68.13%) | 36 (70.59%) | 0.870 |
| Insulin (%) | 110 (40.29%) | 27 (52.94%) | 0.122 |
| Duration of diabetes (years) | 8.01 ± 6.27 | 11.36 ± 7.67 | 0.068 |
| Diabetic complications (%) | 112 (41.03%) | 30 (58.82%) | ***0.021*** * |
| Diabetic Retinopathy (%) | 61 (22.34%) | 18 (35.29%) | 0.053 |
| Diabetic Nephropathy (%) | 26 (9.52%) | 6 (11.76%) | 0.611 |
| Diabetic Peripheral Neuropathy (%) | 48 (17.58%) | 12 (23.53%) | 0.328 |
| Cardiovascular disease (%) | 32 (11.72%) | 11 (21.57%) | 0.071 |
| Hypertension (%) | 140 (51.28%) | 29 (56.86%) | 0.542 |
| Hyperlipidemia (%) | 63 (23.08%) | 11 (21.57%) | 1.000 |
| HbA1c (%) | 7.78 ± 1.75 | 7.98 ± 1.56 | 0.560 |
| FPG (mmol/L) | 8.28 ± 2.81 | 9.45 ± 3.53 | ***0.016*** * |
| MMSE | 28.73 ± 1.06 | 25.22 ± 0.73 | ***<0.001*** ** |
| Olfactory | 6.95 ± 1.72 | 7.74 ± 2.25 | ***0.008*** ** |
| ApoE ε2 | 62 (22.71%) | 10 (19.61%) | 0.716 |
| ApoE ε3 | 252 (92.31%) | 45 (88.24%) | 0.404 |
| ApoE ε4 | 37 (13.55%) | 13 (25.49%) | ***0.036*** * |
| tGSK3β | 1.06 (0.53-1.97) | 1.49 (0.64-2.31) | 0.207 |
| pS9GSK3β | 1.96 (0.77-3.95) | 0.85 (0.36-2.23) | 0.132 |
| rGSK3β | 0.67 (0.36-1.08) | 1.63 (0.84-3.25) | ***<0.001*** ** |

*, p value<0.05; **,p value<0.01.

T2DM, type-2 diabetes mellitus; MCI, mild cognitive impairment; T2DM-NM,T2DM patients remaining with normal cognition; T2DM-CI, T2DM patients progressing to MCI; MMSE, Minimum Mental State Examination; BMI, body mass index; FPG, fasting plasma glucose; HbA1c, glycosylated hemoglobin A1c; ApoE, apolipoprotein E; GSK-3β, glycogen synthase kinase-3β; tGSK-3β, total GSK-3β; pS9GSK-3β, serine-9 phosphorylated GSK-3β; rGSK-3β, total GSK-3β / Ser9 GSK-3β.
