# Supplementary material for: Association of glycogen synthase kinase-3β with cognitive impairment in type 2 diabetes patients: a six-year follow-up study
Source: Front Endocrinol (Lausanne). 2024 Apr 10;15:1386773. doi: 10.3389/fendo.2024.1386773 (PMC11039938; doi:10.3389/fendo.2024.1386773)
Supplement: Supplementary Table 2 — Baseline characteristics of T2DM patients attended and non-attended the follow-up study. [file Table_2.docx]

**Supplementary Table 2. Baseline characteristics of T2DM patients attended and non attended the follow-up study**

|  | **Attenders** | **Non-Attenders** |  |
| --- | --- | --- | --- |
| **Characteristics** | **(n=248)** | **(n=25)** | **P value** |
| Age(years) | 63.10 ± 7.39 | 67.24 ± 10.98 | 0.076 |
| Male (%) | 106 (42.74%) | 11 (44.00%) | 1.000 |
| BMI (kg/m²) | 24.32 ± 2.98 | 24.23 ± 2.60 | 0.888 |
| Cigarette smoking (%) | 39 (15.73%) | 3 (12.00%) | 0.777 |
| Habitual alcohol drinking (%) | 20 (8.06%) | 3 (12.00%) | 0.453 |
| **Education** |  |  | 0.828 |
| ≤ 6 years (Primary school) | 38 (15.32%) | 5 (20.00%) |  |
| 7-9 (Middle school) | 167 (67.34%) | 16 (64.00%) |  |
| ≥ 10 years (High school or college) | 43 (17.34%) | 4 (16.00%) |  |
| Oral medication only (%) | 168 (67.74%) | 18 (72.00%) | 0.823 |
| Insulin (%) | 100 (40.32%) | 10 (40.00%) | 1.000 |
| Duration of diabetes (years) | 8.01 ± 6.27 | 11.36 ± 7.67 | 0.357 |
| Diabetic complications (%) | 104 (41.94%) | 8 (32.00%) | 0.398 |
| Diabetic Retinopathy (%) | 57 (22.98%) | 4 (16.00%) | 0.615 |
| Diabetic Nephropathy (%) | 25 (10.08%) | 1 (4.00%) | 0.486 |
| Diabetic Peripheral Neuropathy (%) | 44 (17.74%) | 4 (16.00%) | 1.000 |
| Cardiovascular disease (%) | 28 (11.29%) | 4 (16.00%) | 0.511 |
| Hypertension (%) | 129 (52.02%) | 11 (44.00%) | 0.531 |
| Hyperlipidemia (%) | 54 (21.77%) | 9 (36.00%) | 0.133 |
| HbA1c (%) | 7.73 ± 1.68 | 8.22 ± 2.32 | 0.178 |
| FPG (mmol/L) | 8.24 ± 2.82 | 8.63 ± 2.68 | 0.516 |
| MMSE | 28.77 ± 1.06 | 28.28 ± 1.06 | ***0.027*** * |
| Olfactory | 6.93 ± 1.73 | 7.14 ± 1.58 | 0.568 |
| ApoE ε2 | 56 (22.58%) | 6 (24.00%) | 0.807 |
| ApoE ε3 | 230 (92.74%) | 22 (88.00%) | 0.422 |
| ApoE ε4 | 32 (12.90%) | 5 (20.00%) | 0.354 |
| tGSK3β | 1.04 (0.53,1.91) | 1.19 (0.50,2.35) | 0.483 |
| pS9GSK3β | 2.00 (0.79,3.80) | 1.60 (0.58,3.53) | 0.645 |
| rGSK3β | 0.66 (0.36,1.06) | 0.89 (0.43,1.40) | 0.111 |

*, p value<0.05; **,p value<0.01.

T2DM, type-2 diabetes mellitus; MCI, mild cognitive impairment; T2DM-NM,T2DM patients remaining with normal cognition; T2DM-CI, T2DM patients progressing to MCI; MMSE, Minimum Mental State Examination; BMI, body mass index; FPG, fasting plasma glucose; HbA1c, glycosylated hemoglobin A1c; ApoE, apolipoprotein E; GSK-3β, glycogen synthase kinase-3β; tGSK-3β, total GSK-3β; pS9GSK-3β, serine-9 phosphorylated GSK-3β; rGSK-3β, total GSK-3β / Ser9 GSK-3β.
